# Mitochondrial Mechanisms of Necroptosis in Liver Diseases

**DOI:** 10.3390/ijms22010066

**Published:** 2020-12-23

**Authors:** Chen Xue, Xinyu Gu, Ganglei Li, Zhengyi Bao, Lanjuan Li

**Affiliations:** 1 State Key Laboratory for Diagnosis and Treatment of Infectious Diseases, National Clinical Research Center for Infectious Diseases, Collaborative Innovation Center for Diagnosis and Treatment of Infectious Diseases, The First Affiliated Hospital, College of Medicine, Zhejiang University, Hangzhou 310003, China; 11918229@zju.edu.cn (C.X.); 11918228@zju.edu.cn (X.G.); 11918216@zju.edu.cn (Z.B.); 2 Department of Neurosurgery, The First Affiliated Hospital, College of Medicine, Zhejiang University, Hangzhou 310003, China; 22018429@zju.edu.cn

**Keywords:** mitochondrial, necroptosis, PGAM5, cyclophilin D, ROS, liver diseases

## Abstract

Cell death represents a basic biological paradigm that governs outcomes and long-term sequelae in almost every hepatic disease. Necroptosis is a common form of programmed cell death in the liver. Necroptosis can be activated by ligands of death receptors, which then interact with receptor-interactive protein kinases 1 (RIPK1). RIPK1 mediates receptor interacting receptor-interactive protein kinases 3 (RIPK3) and mixed lineage kinase domain-like protein (MLKL) and necrosome formation. Regarding the molecular mechanisms of mitochondrial-mediated necroptosis, the RIPK1/RIPK3/MLKL necrosome complex can enhance oxidative respiration and generate reactive oxygen species, which can be a crucial factor in the susceptibility of cells to necroptosis. The necrosome complex is also linked to mitochondrial components such as phosphoglycerate mutase family member 5 (PGAM5), metabolic enzymes in the mitochondrial matrix, mitochondrial permeability protein, and cyclophilin D. In this review, we focus on the role of mitochondria-mediated cell necroptosis in acute liver injury, chronic liver diseases, and hepatocellular carcinoma, and its possible translation into clinical applications.

## 1. Introduction

Liver diseases, including hepatitis B, hepatitis C, nonalcoholic fatty liver disease (NAFLD), alcoholic liver disease (ALD) and related hepatic fibrosis, liver cirrhosis, liver failure, and hepatocellular carcinoma (HCC), are the leading causes of illness and death worldwide [1]. Despite extensive research on the pathogenesis of various liver diseases, there are still no effective therapies for end-stage liver disease or targeted therapies for NAFLD and alcoholic liver disease (ALD) [2,3,4]. Thus, a better understanding of the molecular mechanisms and development of potential therapeutic target sites for liver diseases are of great importance.

Mitochondria play a pivotal role in the generation of cellular energy and biosyn-thesis. They are also key in the regulation of various types of cell death, including necroptosis [5,6,7,8], apoptosis [9,10,11], ferroptosis [12,13,14,15], pyroptosis [16], and other forms. Because mitochondria play a crucial role in energy production [17,18], they must withstand most cellular “stressors” including drugs, viruses, and metabolic disorders [19,20,21]. Impaired mitochondria therefore result in a high level of oxidant production, defects in oxidative phosphorylation, or calcium overload, which then become core factors determining cell survival or death [22,23,24].

Necroptosis is a new form of programmed cell death, but it is challenging to detect in vivo. Aberrant levels of necroptosis have been implicated in various inflammatory diseases and ischemic injury, including liver diseases (specifically nonalcoholic fatty liver diseases, nonalcoholic steatohepatitis, and liver cancer) [25,26,27,28,29]. The molecular necrotic pathway is usually reduced by extracellular signals (such as ligation of death receptors) or intracellular cues (such as viral nucleic acids). The best typical signal transduction cascade leading to the cell necroptosis is initiated by tumor necrosis factor receptor (TNFR). The TNFR and other death receptors engage with their ligands. Then the necroptosis occurs through the activating receptor-interactive protein kinases 1 (RIPK1), PIPK3, and subsequently the pseudokinase mixed lineage kinase domain-like protein (MLKL) in certain cell types [30]. Importantly, induction of necroptosis involves mitochondria and reactive oxygen species (ROS) [31]. The necrosome complex (RIPK1/RIPK3/MLKL) was proposed to induce necroptosis via induction of mitochondrial dysfunction involving a variety of mechanisms, including induction of mitophagy [5], production of mitochondrial ROS [8], activation of the mitochondrial phosphatase phosphoglycerate mutase family member 5 (PGAM5) [32,33], or induction of mitochondrial permeability transition (MPT) [34], as well as by other processes [35].

In this review, we describe how mitochondria regulate necroptosis. Specifically, we focus on the role of mitochondria-mediated cell necroptosis in acute liver injury, chronic liver hepatitis, liver cirrhosis, and hepatocellular carcinoma, as well as the possible translations of these processes into clinical applications.

## 2. Mitochondrial Regulation of Necroptosis

Different types of cell death have distinct morphological and biochemical features. Necrosis is unregulated cell death and often involves the release of various pro-inflammatory factors, resulting in obvious inflammation and tissue damage [36]. Apoptosis is characterized by cytoplasmic and nuclear condensation (causing cell shrinkage) and cell membrane blebbing, but unlike necrosis, there is no membrane disruption or cell leakage and, therefore, there is a lack of inflammatory response [37]. Necroptosis is a new form of programmed necrosis, which has features shared with both necrosis and apoptosis, such as being programmed but causing inflammation and tissue damage. Although cell death was initially thought to be the result of inflammation, recent studies increasingly suggest that cell death may occur first and trigger or amplify the inflammatory response [38].

Necroptosis is a regulated caspase-independent form of cell death involved in various physiological and pathophysiological conditions [28,39,40,41,42]. On the one hand, many studies have reported that the necrosome complex, RIPK1/RIPK3/MLKL, induces necrotic death by induction of mitochondrial dysfunction [31,32,43,44]. On the other hand, a variety of studies had questioned the importance of each of the mitochondrial facets in necroptosis.

### 2.1. ROS and the Necroptosis Signaling Pathway

Although the necrosome complex is known to induce necroptosis, its mechanism of action is unclear. Several studies have revealed the association between ROS generation and necrosome signaling [6,7,8].

In some cell types, mitochondrial ROS is essential for necroptosis by facilitating RIPK1 autophosphorylation, RIPK3 recruitment, and necrosome formation [6,7]. In a feedforward manner, RIPK1-mediated mitochondrial dysfunction and RIPK3 kinase activate the pyruvate dehydrogenase complex, leading to excessive levels of ROS [8,45]. Schenk et al. reported that ROS is an important regulator of BV6/tumor necrosis factor-a (TNF-a)-induced necroptotic signaling and cell death. BV6/TNFa-stimulated ROS generation promotes the formation of the necrosome complex, while silencing RIPK1 and RIPK3 reduces ROS production [6]. Vanlangenakker et al. found that the loss of cellular inhibitor of apoptosis protein-1 did not affect the basal level of ROS in L929 cells, but upon TNF stimulation, ROS generation was significantly increased and the effect was inhibited by knocking down RIP3 and Nec-1(RIP1 inhibitor). Their results indicated a requirement for RIP1/RIP3-dependent ROS in the process of necroptosis [46]. Another study showed that ROS induction required RIPK3, and ROS functioned in a positive feedback circuit that ensured effective induction of necroptosis in L929 cells [7]. In addition, to show the execution pathways of necroptosis, Sun et al. showed that, in human colon cancer cells, the RIPK1/RIPK3 complex induced necroptosis by accumulating cytosolic calcium through c-Jun N-terminal kinase (JNK) activation and mitochondrial ROS production [47]. The current data show that in human acute myeloid leukemia HL60 cells, necroptosis mediation is closely associated with mitochondrial ROS levels [48]. Taken together, these results definitively show that the production of ROS from mitochondria is involved in necroptotic signaling during execution. However, TNF-induced ROS production was dependent on mitochondria, and the inhibition of TNF-induced necroptosis by butylated hydroxyanisol was observed in cells of mitochondrial depletion [5]. Further experimental research is needed to show whether the mitochondrial ROS pathway is cell-specific or universal to all cells as an essential component of necroptosis.

### 2.2. Phosphoglycerate Mutase Family Member 5 (PGAM5)

**Figure 1:**
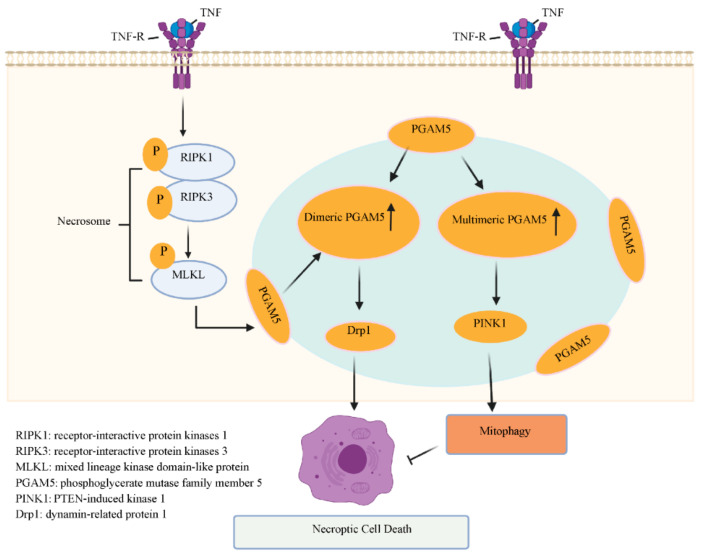
The double function of phosphatase phosphoglycerate mutase family member 5 (PGAM5) in necroptosis. A. After activation of death receptors, tumor necrosis factor signaling can instead induce activation of receptor-interactive protein kinases 1 (RIPK1) and receptor-interactive protein kinases 3 (RIPK3). RIPK3 phosphorylates binds to mixed lineage kinase domain-like protein (MLKL), causing generation of necrosomes. The RIPK1/RIPK3/MLKL necrosome shuttles to the mitochondrial membrane, where RIPK3, after phosphorylation, activates PGAM5 located on the outer membrane of mitochondria. The activated PGAM5 is recruited to mitochondria and activates dynamin-related protein 1, resulting in necroptosis. B. PGAM5/PTEN-induced kinase 1 (PINK1)-mediated mitophagy causes the accumulation of abnormal mitochondria leading to the overproduction of reactive oxygen species, which worsens necroptosis.

Studies have shown that during tumor necrosis factor (TNF)-induced necroptosis, the interaction of RIPK3 with MLKL also induces translocation of the RIPK1/RIPK3/MLK necrosome complex to the mitochondrial membrane, where RIPK3 activates an increasing number of targets by phosphorylation [49,50,51]. One of these targets is PGAM5, an atypical mitochondrial Ser/Thr phosphatase that localizes to the outer membrane of mitochondria with its C-terminus facing the cytoplasm. Two splice variants of PGAM5, PGAM5L and PGAM5S [52,53,54], are recruited to mitochondria, via RIPK3-dependent phosphorylation, in which PGAM5 dephosphorylates and activates dynamin-related protein 1 (Drp1), subsequently leading to necroptosis [32,49] (Figure 1).

It is increasingly clear that PGAM5 is implicated in mitochondrial clustering, fragmentation, mitophagy, apoptosis, and necroptosis [32,50,51,55,56]. Increased accumulation of dimeric PGAM5, with a concomitant reduction in phosphatase activity, not only induces fragmentation of mitochondria, but also sensitizes cells to death signals [57]. Conversely, increased multimeric PGAM5 with a concomitant increase in phosphatase activity induces nuclear clustering of mitochondria, which leads to mitophagy as a cell-protective mechanism for handling damaged mitochondria [57]. To characterize the biological function of PGAM5, Morimarki et al. generated *Pgam5*^−/−^ mice and found that these mice responded normally to multiple inducers of necroptosis, indicating that PGAM5 was dispensable for necroptosis [58]. However, Lu et al. also found that PGAM5 was indispensable for the process of PTEN-induced kinase 1 (PINK1)-dependent mitophagy which antagonizes necroptosis. The loss of PGAM5/PINK1-mediated mitophagy causes the accumulation of abnormal mitochondria, leading to overproduction of ROS, which worsens necroptosis. Taken together, the results suggest that PGAM5 is downstream of RIPK1/RIPK3, induces necroptosis, and protects cells from necroptosis via promoting mitophagy [59] (Figure 1).

### 2.3. Metabolic Enzymes

**Figure 2:**
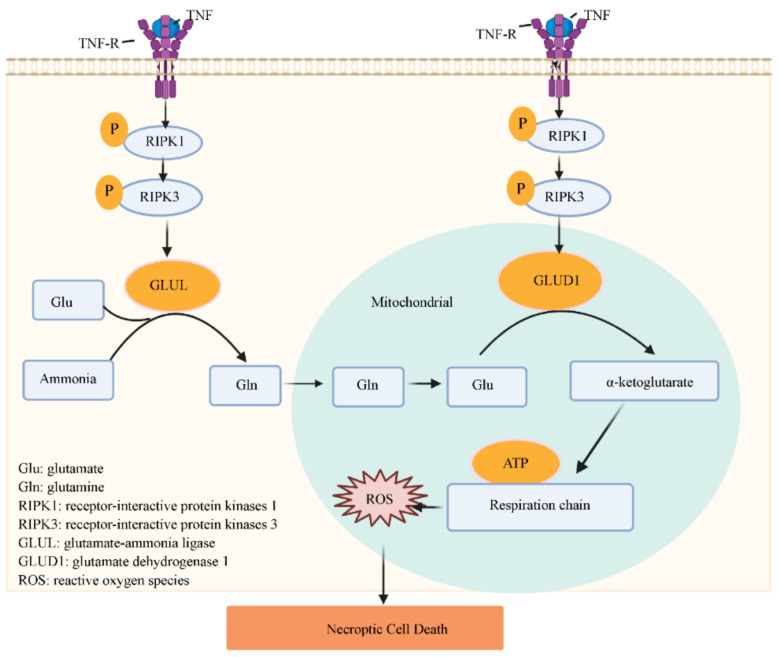
The role of metabolic enzymes of mitochondria in necroptosis. Endogenous RIPK3 can directly increase glutamate-ammonia ligase (GLUL) and glutamate dehydrogenase 1 (GLUD1) activity. GLUL is a cytosolic enzyme that catalyzes the condensation of glutamate and ammonia to form glutamine. Glutamine can translocate into the mitochondria to function as an energy substrate. GLUD1 is a mitochondrial matrix enzyme that converts glutamate to α-ketoglutarate. GLUL and GLUD1-mediated uses of glutamate or glutamine as energy substrates contribute to necroptosis.

There is also evidence that some metabolic enzymes in mitochondria are involved in TNF-induced necroptosis [60,61]. Zhang et al. reported that endogenous RIPK3 directly increased glutamate-ammonia ligase (GLUL) and glutamate dehydrogenase 1 (GLUD1) activity. GLUL is a cytosolic enzyme that catalyzes the condensation of glutamate (Glu) and ammonia to form glutamine (Gln), which can be translocated into mitochondria to function as an energy substrate [60,62]. GLUD1 is a mitochondrial matrix enzyme that converts Glu to α-ketoglutarate, which provides the energy substrates for energy metabolism–associated ROS production [61,63]. Silencing GLUL or GLUD1 using siRNA attenuates TNF-induced necroptosis in NIH 3T3 cells, suggesting that GLUL and GLUD1 mediate the use of Glu or Gln as energy substrates, which contributes to necroptosis [60,64] (Figure 2).

### 2.4. The mPTP and Cyclophilin-D

Mitochondrial permeability transition pores (mPTPs) may be another potential mitochondrial mediator of necroptosis. The mPTP is a nonspecific channel that spans the inner mitochondrial membrane. Organ injury results in the opening of mPTPs, which leads to changes in mitochondrial transmembrane potentials, dysfunction of oxidative phosphorylation, accumulated ROS, and ultimately mitochondrial rupture [65]. Inhibition of the mPTP opening suppresses necroptotic cell death, suggesting the involvement of mPTPs in the necroptotic pathway. In addition, cyclophilin-D is an important regulator of mPTPs, and can control the process of necroptosis [33,34]. Cyclophilin-D knockdown protected mouse microvascular endothelial cells from necroptosis by inhibiting RIPK3-downstream mix-lineage kinase domain-like protein phosphorylation [34]. He et al. reported that loss of cyclophilin-D reduced necroptosis in mouse embryonic fibroblasts [66], and another study reported that a cyclophilin-D inhibitor inhibited TNF-induced zebrafish macrophage ROS accumulation and necroptosis [33].

### 2.5. The B Cell Lymphoma 2 (BCL-2) Family

The BCL-2 protein family comprises three subsets: anti-apoptotic proteins (BCL-2, MCL-1, BCL-W, and BCL-X_L_), pro-apoptotic BCL-2 proteins (BAK, BAX, and BOK) and BH3-only proteins (BID, BIM, BAD, BMF, HRK, PUMA, NOXA, and BIK). Present studies have demonstrated that the BCL-2 family members induced outer membrane permeabilization to mediate cell apoptosis [67,68,69]. Recently, Karch et al. reported that BCL-2 family members are also required for mitochondrial pore-dependent necrotic cell death by facilitating outer membrane permeability of the MPTP [70]. Absence of *Bax*/*Bak* decreased the outer mitochondrial membrane permeability without altering the inner membrane MPTP function, leading to resistance to mitochondrial calcium overload and necroptosis.

Hitomi et al. identified the BMF protein of the BCL2 family in the mitochondrial outer membrane as a potential mediator of TNF-α -induced necroptosis [71]. At least in L929 cells, knockdown of *BMF* suppressed the necrotic response to TNFα. Whether the same mechanism exists in other types of cells is unknown, and the function of BMF in mitochondria has not been definitively established [71]. Lin et al. found that overexpression of *BCL-2* alleviated cytochrome c release and necroptosis induced by green tea polyphenols, and knockdown of *BAX* and *BAK* in Hep3B cells also ameliorated cytochrome c release and necroptosis [72]. These results indicate that necroptosis was related to the translocation of BAX and BAK to the mitochondria and the release of cytochrome c [72]. BAX/BAK were implicated as necessary mediators of necroptosis [35,73]. However, other studies suggested that the BCL-2 family has no involvement [5,74].

### 2.6. Others

Many studies have described the pivotal role of mitochondria in execution of the necroptotic progress. However, several studies have questioned the importance of each of the mitochondrial facets in necroptosis [5,43,75,76,77]. It has also been shown that cells depleted of mitochondria through enforced mitophagy remain able to undergo necroptosis, implying that mitochondria or mitochondrial metabolism are not essential for the execution of necroptosis [5].

## 3. Mitochondrial Mechanisms of Necroptosis in Liver Diseases

### 3.1. Acute Liver Injury

Mitochondrial dysfunction has also been linked to acute liver injury. Atypical mitochondrial Ser/Thr phosphatase PGAM5 is overexpressed in hepatocytes of patients with autoimmune hepatitis and in mice with ConA-induced experimental hepatitis [78]. Furthermore, silencing PGAM5 protects mice from ConA-induced hepatocellular death [78]. Qian et al. reported that the RIPK1/RIPK3/MLKL signaling pathway was activated in mice with acute liver injury induced by *Listeria monocytogenes* infection, subsequently leading to necroptosis and hepatic damage. Knockdown of RIPK1 attenuates mitochondrial dysfunction and necroptosis in hepatic tissues from *L. monocytogenes*-infected mice [79]. Some research groups have reported that RIP1 and RIP3 are both critical mediators of necroptosis in APAP-induced acute liver injury [71,80]. The RIP1 inhibitor not only suppresses APAP-induced translocation of BAX from the cytoplasm to mitochondria, but also suppresses APAP-induced translocation of AIF from the mitochondria to nuclei, suggesting that mitochondrial Bax and AIF translocations might be key events in the process of APAP-induced necroptosis [81].

### 3.2. Chronic Liver Diseases

Cell death is an important feature in chronic liver disease, and apoptosis is the predominant type of cell death observed. A great deal of research revealed an unequivocal link between cell apoptosis and chronic liver diseases [82,83], while the occurrence of necroptosis in the liver and its contribution to the chronic liver diseases is controversial [84]. Present studies showed that activated MLKL forms translocate to the cell membrane and execute necroptosis [77,85,86]. MLKL-driven rupture of the cell membrane is the necessary step of necroptosis. At present, the only known activator of MLKL is RIPK3. Thus, MLKL and RIPK3 are necessary for necroptotic death [87]. Notably, hepatocytes clearly express MLKL, while they do not express RIPK3 under basal conditions [88]. RIPK3 was not detected even in primary mouse hepatocytes after cell culture with TNF or APAP at different time points [88,89]. Whether hepatocytes express RIPK3 or not is crucial, because as far as we know, hepatocytes that do not express RIPK3 will not undergo necroptosis [90]. However, in some unique circumstances, for example, MLKL contributes to hepatocyte death in concanavalin A injury and that MLKL-induced death is independent of the PIPK3 [91]. Roychowdhury et al. examined the effects of RIPK3 using an alcoholic model [92]. They reported that increased expression of RIP3 was found in liver tissues of mice after chronic ethanol feeding, as well as in liver tissues from patients with alcoholic liver disease. The *RIP3*^−/−^ mice were protected from ethanol-induced steatosis, hepatocyte injury, and expression of proinflammatory cytokines compared with WT (C57BL6/j) mice. While the expression of RIP1 in mouse liver tissues had no change following ethanol feeding, and inhibition of RIP1 kinase by necrostatin-1 did not mitigate ethanol-induced hepatocyte injury [92]. Roychowdhury et al. also explored the contribution of necroptosis to high fat diets (HFD)-induced liver injury [93]. They found the expression levels of RIPK3 and MLKL were increased in HFD-fed C57BL/6 mice liver. HFD did not increase MLKL in RIPK3 knockout mice. The *RIPK3*^−/−^ mice had basic glucose intolerance even on chow diets. Interestingly, HFD worsened the hepatic steatosis, inflammation, and hepatocellular apoptosis in *RIPK3*^−/−^ mice compared with WT control [93]. *RIPK3^−/−^* had diametrically opposite results in the HFD model and the alcohol model.

Although the proximal molecular pathway of the necroptosis is well studied, the downstream signaling has been poorly understood. Recent advances in our understanding of the role of mitochondria have led to the recognition that impaired mitochondrial function may be responsible for chronic liver diseases [21,94,95,96]. The relationships between mitochondria and necroptosis in chronic liver disease are poor studied.

### 3.3. Hepatocellular Carcinoma (HCC)

Increasing evidence has shown that necroptosis occurs in hepatoma cells through mitochondrial-associated signaling pathways [97,98,99]. Heslop et al. reported that sorafenib promotes hepatoma cell death by necroptosis and induces mitochondrial dysfunction [97]. Moreover, activated JNK translocation to mitochondria blocks electron transport at Complex I or II, thereby decreasing the oxidative phosphorylation system (OXPHOS) and promoting ROS formation as a common mechanism of mitochondrial dysfunction [98]. Cell necroptosis is suppressed by a JNK inhibition, indicating that mitochondrial dysfunction promoted by JNK is an important driver of necroptosis [97]. In another study, BAX and BAK, two essential mitochondrial permeability transition pore proteins, were activated by green tea polyphenols and by their translocation and homo-oligomerization in mitochondria. Necroptosis was ameliorated in Hep3B cells (*BAK*^−/−^), and Hep3B cells (*BAX*^−/−^). Moreover, overexpression of BCL-2 decreased necroptosis. The results suggested that necroptosis could be induced by green tea polyphenols in p53-deficient Hep3B cells, and that necroptosis was associated with BAX/BAK translocation and homo-oligomerization in mitochondria [72]. Rizza et al. found that knockdown of S-nitrosoglutathione reductase resulted in mitochondrial defects and that S-nitrosoglutathione reductase deficient HepG2 cells and tumors were sensitive to succinate dehydrogenase inhibition, which induced RIP1/PARP1-mediated necroptosis and suppressed tumor growth [99].

## 4. Therapies and Perspectives

Mitochondria play an essential role in necroptosis, suggesting that efficient strategies to target mitochondrial-induced cell death pathways may have particular promise for liver disease. Majdi et al. reported that the necroptosis pathway was activated during nonalcoholic fatty liver disease, and inhibition of RIPK1 ameliorated the characteristics of non-alcoholic steatohepatitis in high fat diet fed mice [100]. Furthermore, it reversed steatosis via an MLKL-dependent mechanism, which was at least partially involved in mitochondrial respiration [100]. A recent study showed that targeting nuclear protein 1-induced cell death and controlling HCC progress by apoptosis and necroptosis led to mitochondrial metabolism failure via inhibiting intracellular levels of ATP in HepG2 and HepB3 cells [101]. Prevention of mitochondrial alterations promises to be an effective strategy for treatment of in liver inflammatory diseases and HCC. However, at present, no anti-mitochondrial drugs have proven to be absolutely effective. New selective molecules that target mitochondrial dysfunction are needed for different liver pathology.

A current drawback is that animal models used to study mechanisms of liver diseases lack the full features of the human disease [102,103,104]. Some existing strategies have been reported to target mitochondrial dysfunction with liver diseases, but they are likely to have many nonspecific effects [105,106,107]. It will therefore be important to develop and test small molecules that target specific steps in the mitochondrial-mediated cell death signaling pathways. Advances in gene manipulation technology could enable the correction of mutated genes important in mitochondrial-mediated cell death pathways, thereby altering the course of certain liver disorders.

## 5. Conclusions

Mitochondrial function and cell death pathways have long been considered to be two of the major regulators of cell survival or death. With identification of a newer cell death pathway, necroptosis has been extensively studied in different hepatic disease models. Notably, mitochondria not only control energy production, oxidative phosphorylation, and ROS generation, but also participate in the necroptosis pathway by regulating translocation of Bax and Bak to mitochondria and multiple mitochondrial constituents (PGAM5, cyclophilind-D, and some metabolic enzymes). In various liver diseases, mitochondria are multifaceted regulators of the necroptosis pathway to promote inflammation, enhance immune responses, and regulate the progression of diseases. In a feedforward manner, necroptosis can lead to mitochondrial dysfunction via the PIPK1/PIPK3/MLKL pathway. Some existing strategies have been discovered, which target mitochondrial dysfunction during liver disease. Advances in gene manipulation technology can enable the correction of mutated genes important for mitochondrial-mediated pathways, thereby altering the course of certain liver disorders. In conclusion, improving our understanding of the dysfunctions involving mitochondrial and mitochondrial-mediated necroptosis could provide potential therapeutic targets for treating liver diseases; however, much work needs to be done before identifying safe and effective inhibitors or drugs that improve mitochondrial function.

## Abbreviations

RIPK1	receptor-interactive protein kinases 1
RIPK3	receptor-interactive protein kinases 3
MLKL	mixed lineage kinase domain-like protein
PGAM5	phosphoglycerate mutase family member 5
NAFLD	nonalcoholic fatty liver disease
ALD	alcoholic liver disease
HCC	hepatocellular carcinoma
ALD	alcoholic liver disease
TNFR	tumor necrosis factor receptor
TNF-α	tumor necrosis factor alpha
ROS	reactive oxygen species
mPTPs	mitochondrial permeability transition pores
BCL-2	B cell lymphoma 2
Drp1	dynamin-related protein 1
PINK1	PTEN-induced kinase 1
GLUL	glutamate-ammonia ligase
GLUD1	glutamate dehydrogenase 1
OXPHOS	oxidative phosphorylation system
